# Efficacy and Safety of Oncolytic Viruses in Randomized Controlled Trials: A Systematic Review and Meta-Analysis

**DOI:** 10.3390/cancers12061416

**Published:** 2020-05-30

**Authors:** Zengbin Li, Zeju Jiang, Yingxuan Zhang, Xiaotian Huang, Qiong Liu

**Affiliations:** 1 Department of Medical Microbiology, School of Medicine, Nanchang University, Nanchang 330006, China; 6300616180@email.ncu.edu.cn (Z.J.); 6300817334@email.ncu.edu.cn (Y.Z.); xthuang@ncu.edu.cn (X.H.); 2 School of Public Health, Nanchang University, Nanchang 330006, China; 3 Key Laboratory of Tumor Pathogenesis and Molecular Pathology, School of Medicine, Nanchang University, Nanchang 330006, China

**Keywords:** oncolytic viruses, oncolytic virotherapy, efficacy, adverse events, systematic review, meta-analysis

## Abstract

Oncolytic virotherapy is a promising antitumor therapeutic strategy. It is based on the ability of viruses to selectively kill cancer cells and induce host antitumor immune responses. However, the clinical outcomes of oncolytic viruses (OVs) vary widely. Therefore, we performed a meta-analysis to illustrate the efficacy and safety of oncolytic viruses. The Cochrane Library, PubMed, and EMBASE databases were searched for randomized controlled trials (RCTs) published up to 31 January 2020. The data for objective response rate (ORR), overall survival (OS), progression-free survival (PFS), and adverse events (AEs) were independently extracted by two investigators from 11 studies that met the inclusion criteria. In subgroup analyses, the objective response rate benefit was observed in patients treated with oncolytic DNA viruses (odds ratio (OR) = 4.05; 95% confidence interval (CI): 1.96–8.33; *p* = 0.0002), but not in those treated with oncolytic RNA viruses (OR = 1.00, 95% CI: 0.66–1.52, *p* = 0.99). Moreover, the intratumoral injection arm yielded a statistically significant improvement (OR = 4.05, 95% CI: 1.96–8.33, *p* = 0.0002), but no such improvement was observed for the intravenous injection arm (OR = 1.00, 95% CI: 0.66–1.52, *p* = 0.99). Among the five OVs investigated in RCTs, only talimogene laherparepvec (T-VEC) effectively prolonged the OS of patients (hazard ratio (HR), 0.79; 95% CI: 0.63–0.99; *p* = 0.04). None of the oncolytic virotherapies improved the PFS (HR = 1.00, 95% CI: 0.85–1.19, *p* = 0.96). Notably, the pooled rate of severe AEs (grade ≥3) was higher for the oncolytic virotherapy group (39%) compared with the control group (27%) (risk difference (RD), 12%; risk ratio (RR), 1.44; 95% CI: 1.17–1.78; *p* = 0.0006). This review offers a reference for fundamental research and clinical treatment of oncolytic viruses. Further randomized controlled trials are needed to verify these results.

## 1. Introduction

Cancer is a common disease globally that seriously affects human health. The USA, for instance, projects to have 1,806,590 and 606,520 new cancer cases and cancer deaths, respectively, in 2020 [1]. Although traditional treatment methods such as radiotherapy, chemotherapy, and targeted drugs are preferred in cancer treatment, their disadvantages include severe adverse events, development of drug resistance, and cross-resistance [2,3]. Therefore, the development of more effective cancer treatment strategies is urgently needed. Oncolytic viruses (OVs) are natural or artificially modified viruses that selectively replicate in and destroy cancer cells; hence, they represent a promising approach for antitumor therapy [4,5]. Oncolytic viruses generally exert antitumor effects by two mechanisms, namely, the selective killing of tumor cells, and induction of antitumor immunity [6]. To achieve specificity for tumor cells, key proteins required by OVs to infect the host are first modified to reduce infection of normal tissues [7,8,9]. Besides, oncolytic viruses utilize signaling pathways such as p53, epidermal growth factor receptor (EGFR)/Ras, and protein kinase R (PKR) to target tumor cells for selective expansion [10,11,12,13]. OVs can also kill tumor cells by triggering the expression of the suicide gene [14,15]. The key steps employed by OVs to transform “cold tumors” into “hot tumors” and activate antitumor immune responses include targeted replication, the release of tumor-associated antigens through oncolysis, upregulation of chemokines and danger signals, recruitment of dendritic cells and lymphoid cells, and upregulation of immune checkpoint molecules [16,17,18].

Oncolytic viruses are either RNA or DNA viruses. RNA viruses such as reoviruses, paramyxoviruses, and picornaviruses, which encode only a few genes, often undergo rapid proliferation and lysis of tumor cells [5,18,19,20]. On the other hand, oncolytic DNA viruses such as herpes viruses, adenovirus, or poxviruses allow for the insertion of multiple foreign genes but are slower in replication and amplification [5,21,22]. The structure, gene components, expression strategies, and antineoplastic mechanisms are therefore different between the two types [23]. Talimogene laherparepvec (T-VEC), which is an oncolytic herpes virus type I, is presently the only oncolytic virus approved by the Food and Drug Administration. The success of T-VEC in the treatment of melanoma has further promoted the research of oncolytic viruses. With the increased number of clinical studies on oncolytic viruses, the efficacy and safety of oncolytic viruses have drawn much attention. Clinical trials of oncolytic viruses in combination with chemotherapeutic drugs, radiotherapy, and immune checkpoint inhibitors have shown massive progress in cancer treatment [5,16,24]. In particular, the combination of oncolytic virus and immune checkpoint inhibitors has yielded good results in melanoma [25]. Although many oncolytic viruses exist, a real champion among the oncolytic viruses has not yet emerged. In addition, no systematic review has been conducted on the efficacy and safety of oncolytic viruses in randomized controlled trials.

In this meta-analysis, we included the following viruses: T-VEC (herpes virus) [26,27], pelareorep (reovirus) [28,29,30,31,32,33], NTX-010 (seneca valley virus; picornavirus) [19], Ad5-yCD/*mut*TK_SR39_*rep*-ADP (adenovirus) [34], and pexastimogene devacirepvec (Pexa-Vec; poxvirus) [35]. We first evaluated the efficacy of oncolytic virus from objective response rate (ORR), overall survival (OS), and progression-free survival (PFS); then we analyzed severe adverse events (grade ≥3) and detailed adverse events (AEs). Overall, we conducted this meta-analysis to investigate the effectiveness and safety of oncolytic viruses in randomized controlled trials to provide insights for fundamental research and clinical treatment.

## 2. Methods

### 2.1. Literature Search Strategy 

A systematic search was conducted in EMBASE, PubMed, and Cochrane databases for studies published up to 30/1/2020. The search terms included: “oncolytic viruses”, or “viruses, oncolytic”, or “oncolytic virus”, or “virus, oncolytic”, or “oncolytic virotherapy”, or “oncolytic virotherapies”, or “virotherapies, oncolytic”, or “virotherapy, oncolytic”, or “oncolytic virus therapy”, or “oncolytic virus therapies”, or “therapies, oncolytic virus”, or “therapy, oncolytic virus”, or “virus therapies, oncolytic”, or “virus therapy, oncolytic”. There was a language restriction of English in the search, and we followed the PRISMA guidelines for randomized controlled trials (RCTs) to conduct the meta-analysis [36].

### 2.2. Inclusion and Exclusion Criteria

We included studies in the meta-analysis if they met the following inclusion criteria: (1) the studies were randomized controlled trials in cancer patients treated with an oncolytic virus; (2) the articles had at least one of the following outcomes: objective response rate (ORR), overall survival (OS), progression-free survival (PFS), or adverse events (AEs); (3) cancer patients in the control group received the control regimen without oncolytic virus. However, articles were excluded if: (1) they were conference abstracts, case reports, letters, meta-analyses, cohort studies, single-arm studies, reviews, animal studies, or in vitro studies; (2) patients in the control group received oncolytic virotherapy; (3) they included literatures with overlapping patients. Two independent investigators screened the potentially eligible articles by reading the titles and abstracts. Thereafter, the full text of all remaining studies was read to determine if they met the set eligibility criteria. Disagreements on study selection were resolved by discussion with other investigators.

### 2.3. Data Extraction

Two investigators independently read full texts of the included literatures and extracted the data. Any divergence of opinions concerning the extracted data was resolved through consultation. The extracted data included first author, publication, year, country, treatment, injection mode of OVs, types of cancer, the total number of patients, and clinical endpoints. The primary endpoints were ORR, OS, and PFS, while secondary endpoints included adverse events, which were evaluated using the National Cancer Institute—Common Terminology Criteria for Adverse Events (version 3.0 or 4.0). In addition, we carefully read supplementary materials of the included literatures to prevent any loss of information.

### 2.4. Quality Assessment

Quality assessment was done by two independent investigators using the Cochrane risk of bias tool. The risk of bias parameters included the random sequence generation (selection bias), allocation concealment (selection bias), blinding of participants and personnel (performance bias), blinding of outcome assessment (detection bias), incomplete outcome data (attrition bias), selective reporting (reporting bias), and other bias. Each entry was determined as high-risk, low-risk, or unclear. If an item could not be assessed due to lack of information, it was considered as having an unclear risk of bias. Disagreements on quality assessment were resolved by consensus.

### 2.5. Statistical Analysis

Statistical analyses were performed using Review Manager (RevMan) 5.3 software and STATA 12.0. Results were presented as hazard ratios (HRs), risk ratios (RRs), or odds ratios (ORs) with 95% CI (confidence interval). Heterogeneity among RCTs was assessed by the Chi-square test and index of heterogeneity (*I*^2^). A mixed-effects model was used when heterogeneity was not significant (*I*^2^ < 50% or *p*-value > 0.1); otherwise, the random-effects model was performed. Publication bias was evaluated statistically via funnel plots, Begg’s test, and Egger’s test. Statistical significance was set at *p* < 0.05. 

## 3. Results

### 3.1. Systematic Review Process and Quality Assessment

**Figure 1:**
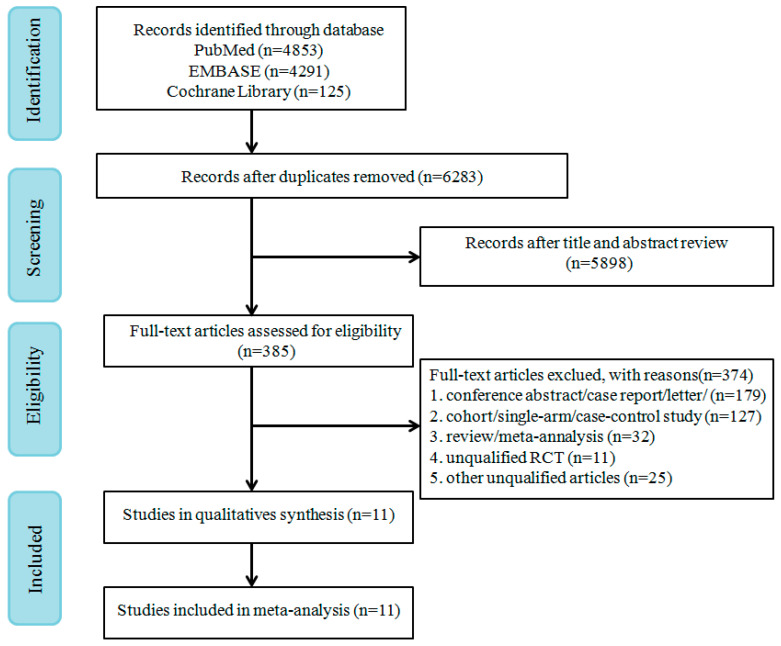
PRISMA flow diagram of randomized controlled trials (RCTs) of patients treated with oncolytic virus.

A total of 9269 records were retrieved from PubMed, EMBASE, and Cochrane Library. A flow chart of study screenings and the election process is shown in Figure 1. From the remaining 6283 references screened after removing duplicates, 385 potentially eligible references were identified. Eventually, 11 RCTs that met the inclusion criteria were selected for full-text review. 

**Figure 2:**
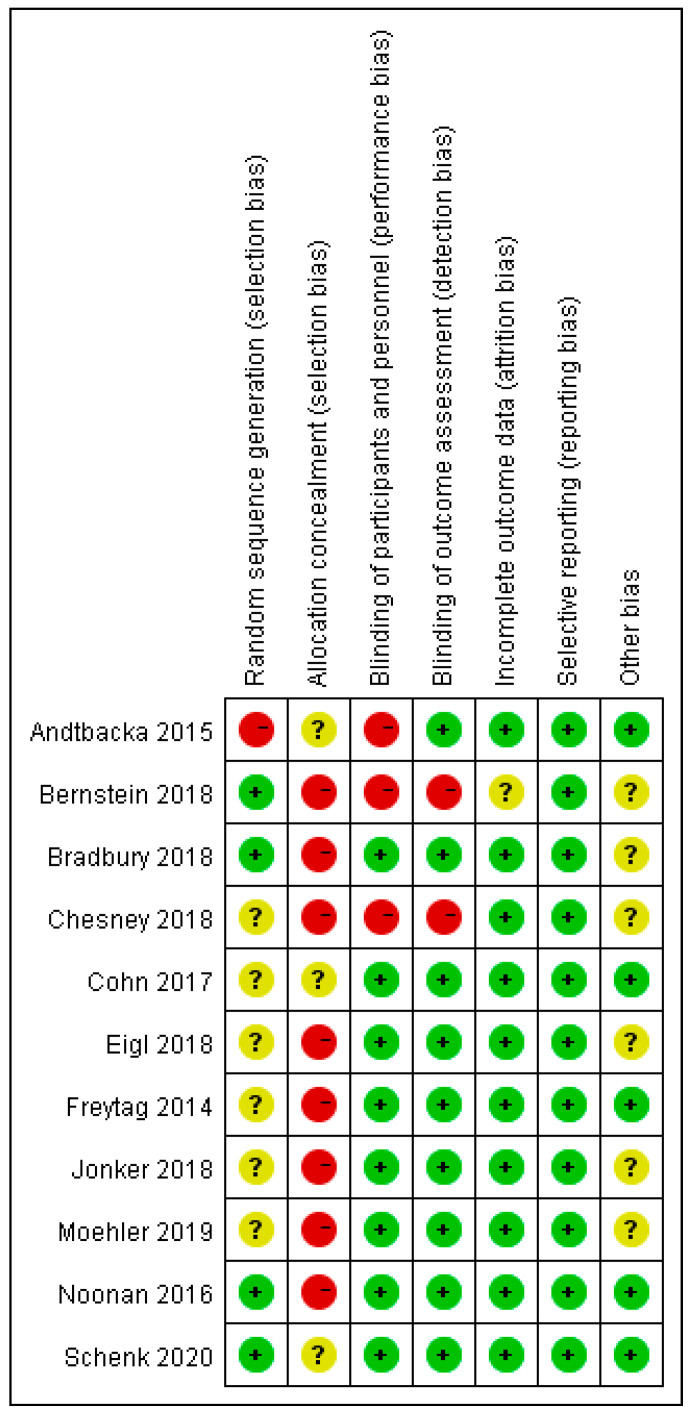
Assessment of risk of bias for 11 included randomized controlled trials.

The risk of bias for the 11 included RCTs is shown in Figure 2. All the included RCTs were open-label trials. Most RCTs mentioned random allocation performed without using the random sequence generation method. Blinding was not performed because of the moral risk associated with the sham injection. In some RCTs [19,29,30,31,32,33,34,35], non-blinding had no significant effect on the efficacy or safety of oncolytic viruses; hence, they were judged as a low-risk factor.

### 3.2. Characteristics of Studies

We included eleven studies with a total of 1452 patients in this meta-analysis. The characteristics and outcomes of RCTs are presented in Table 1 and Table 2. The OVs used in the included trials were T-VEC (*n* = 2), pelareorep (*n* = 6), NTX-010 (*n* = 1), Ad5-yCD/mutTKSR39rep-ADP (*n* = 1), and Pexa-Vec (*n* = 1). The types of tumors included melanoma, breast cancer, lung cancer, prostate cancer, hepatocellular carcinoma, colorectal cancer, pancreatic adenocarcinoma, and ovarian, tubal, or peritoneal cancer. The injection methods were either intratumoral or intravenous. Eleven included clinical trials of oncolytic viruses were conducted in the United States and Canada. 

Oncolytic DNA viruses include T-VEC, Pexa-Vec, and Ad5-yCD/mutTKSR39rep-ADP, and they all carry transgenes. T-VEC is modified by deleting the *ICP47* gene and *ICP34.5* gene (the herpes virus neurovirulence factor) to reduce viral pathogenicity and enhance selective tumor replication [37,38]. In addition, T-VEC could elicit human granulocyte macrophage colony-stimulating factor (GM-CSF) to recruit and activate antigen-presenting cells with subsequent induction of tumor-specific T-cell responses [13]. Pexa-Vec (JX-594) is a thymidine kinase gene-inactivated vaccinia virus engineered by expressing the transgenes, including GM-CSF and β-galactosidase; it selectively targets tumor cells with activation of the Ras/MAPK signaling pathway [35,39]. Ad5-yCD/mutTKSR39rep-ADP is adenovirus carrying two cytotoxic gene systems, cytosine deaminase (cytosine deaminase (CD)/5-fluorocytosine (5-FC) and herpes simplex virus thymidine kinase (HSV-1 TK)/valganciclovir (vGCV), and it can enhance the sensitivity of tumor cells to specific drugs and radiation [34].

Oncolytic RNA viruses include pelareorep and NTX-010. Pelareorep is a human reovirus type 3 Dearing strain, which contains live, replication-competent reovirus, and has specific oncolysis with an activated Ras pathway [31,33]. Direct oncolysis of pelareorep led to release of “danger signals”, such as soluble tumor-associated antigens, viral pathogen-associated molecular patterns, and cell-derived damage-associated molecular patterns [16,40]. Therefore, direct oncolysis could result in generating innate and adaptive immune response to the tumor microenvironment and induces the antitumor immune response. Besides, NTX-010 (seneca valley virus) was a novel oncolytic picornavirus, which could target and lyse tumor cells [19,41].

### 3.3. Effectiveness

#### 3.3.1. Objective Response Rate

**Figure 3:**
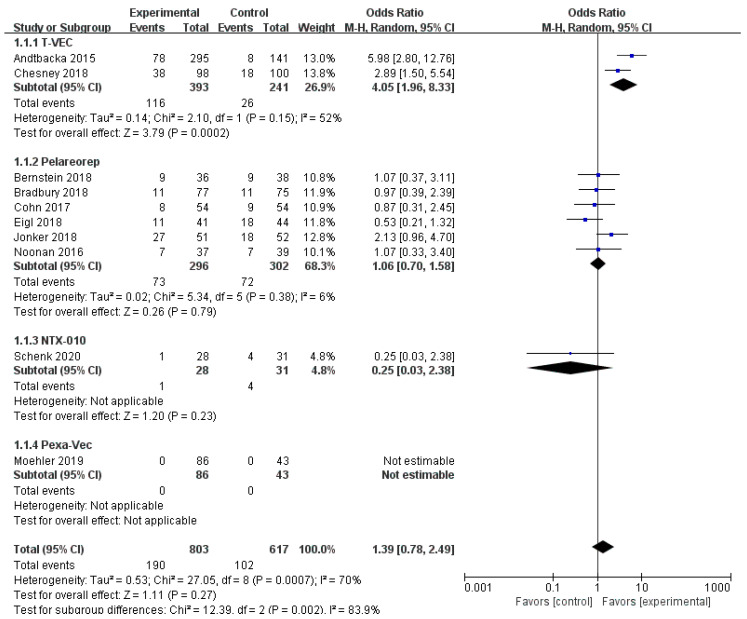
Forest plot of the pooled odds ratios (ORs) for objective response rate (ORR) in different oncolytic virus species.

**Figure 4:**
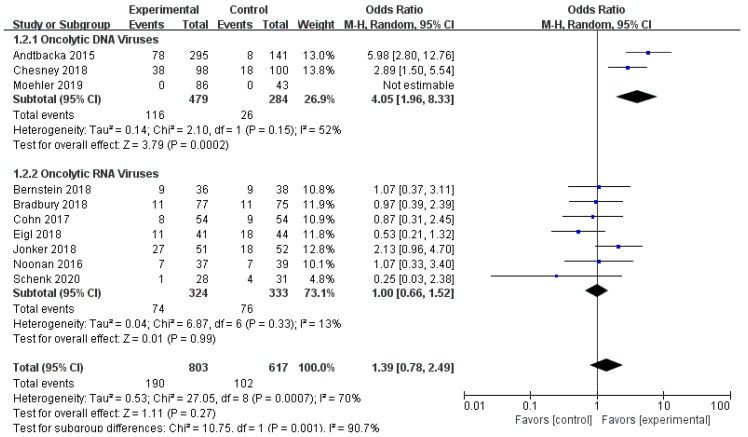
Forest plot of the pooled odds ratios (ORs) for objective response rate (ORR) of oncolytic DNA viruses and oncolytic RNA viruses.

**Figure 5:**
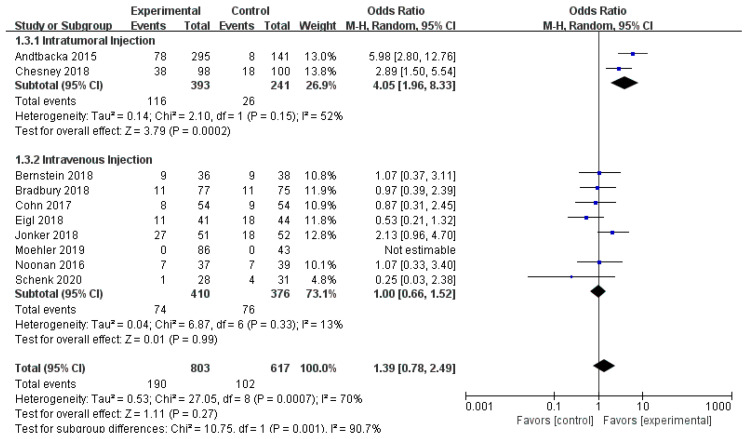
Forest plot of the pooled odds ratios (ORs) for objective response rate (ORR) of intratumoral and intravenous injections.

Ten RCTs reported objective response rate (ORR). Since differences were observed in efficacy among various OVs; we performed subgroup analysis on the ORR based on species, oncolytic DNA/RNA viruses, and injection mode. There was a statistically significant difference in ORRs between patients that received T-VEC (*n* = 2, OR = 4.05, 95% CI: 1.96–8.33, *I*^2^ = 52%, *p* = 0.0002). However, there was no significant difference in ORRs between patients treated with pelareorep (*n* = 6, OR = 1.06, 95% CI: 0.70–1.58, *I*^2^ = 6%, *p* = 0.79), NTX-010 (*n* = 1, OR = 0.25, 95% CI: 0.03–2.38, *p* = 0.23), and Pexa-Vec (*n* = 1, not estimable) (Figure 3). Objective response rate benefit was observed in patients that received oncolytic DNA viruses (*n* = 3, OR = 4.05, 95% CI: 1.96–8.33, *I*^2^ = 52%, *p* = 0.0002) but not in those treated with oncolytic RNA viruses (*n* = 7, OR = 1.00, 95% CI: 0.66–1.52, *I*^2^ = 13%, *p* = 0.99) (Figure 4). In the subgroup analysis for injection methods, results showed that the intratumoral injection arm produced significant improvement (*n* = 2, OR = 4.05, 95% CI: 1.96–8.33, *I*^2^ = 52%, *p* = 0.0002), but no significant improvement was found for the intravenous injection arm (*n* = 7, OR = 1.00, 95% CI: 0.66–1.52, *I*^2^ = 13%, *p* = 0.99) (Figure 5).

#### 3.3.2. Overall Survival and Progression-Free Survival

**Figure 6:**
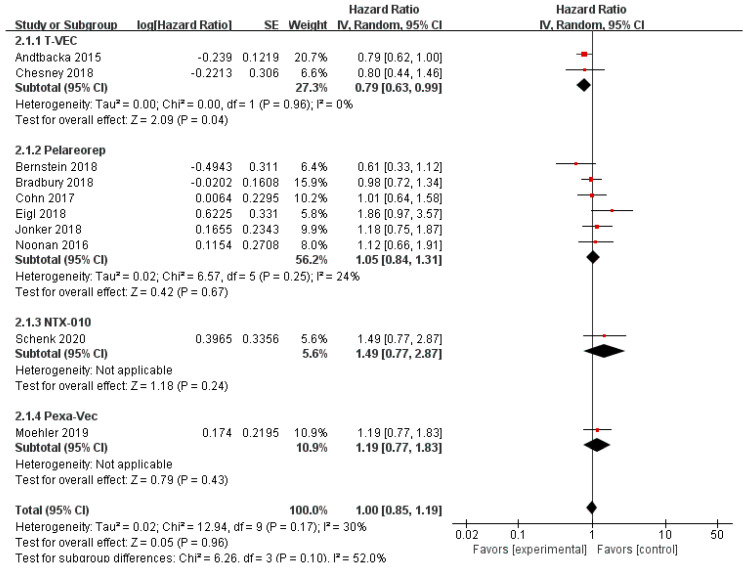
Forest plot of the pooled hazard ratios (HR) for overall survival (OS).

**Figure 7:**
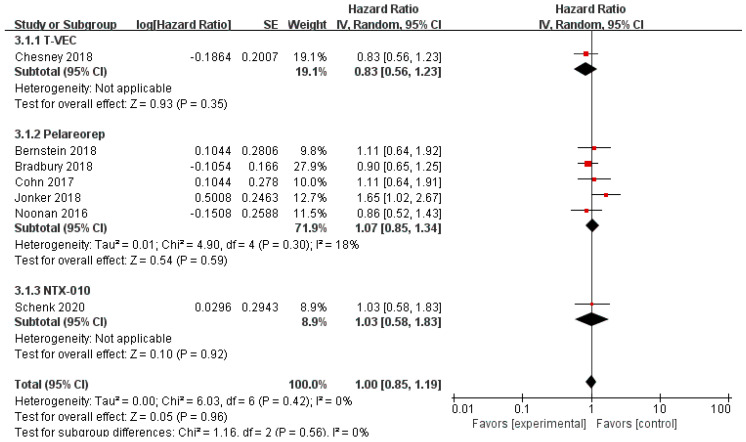
Forest plot of the pooled hazard ratios (HR) for progression-free survival (PFS).

Data regarding overall survival (OS) were available in ten RCTs, seven of which provided data for progression-free survival (PFS). Compared with the control group, patients treated with T-VEC had better OS (*n* = 2, HR = 0.79, 95% CI: 0.63–0.99, *p* = 0.04). However, treatment with pelareorep (*n* = 6, HR = 1.05, 95% CI: 0.84–1.31, *p* = 0.67), Pexa-Vec (*n* = 1, HR = 1.19, 95% CI: 0.77–1.83, *p* = 0.43), and NTX-010 (*n* = 1, HR = 1.49, 95% CI: 0.77–2.87, *p* = 0.24) did not improve the OS significantly compared to the control group (Figure 6). In addition, none of the patients benefited from T-VEC (*n* = 1, HR = 0.83, 95% CI: 0.56–1.23, *p* = 0.35), pelareorep (*n* = 5, HR = 1.07, 95% CI: 0.85–1.34, *p* = 0.59), and NTX-010 treatment (*n* = 1, HR = 1.03, 95% CI: 0.58–1.83, *p* = 0.92) in terms of PFS (Figure 7).

#### 3.3.3. Safety

**Figure 8:**
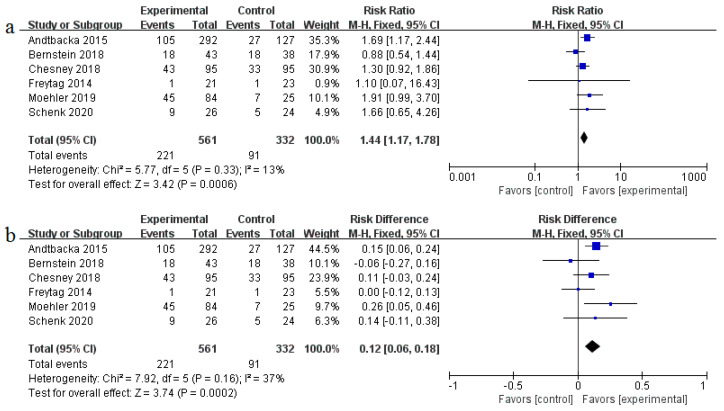
Forest plot of severe adverse events (grade ≥3): (**a**) the pooled risk ratios (RR); (**b**) the pooled risk difference (RD).

Safety of oncolytic viruses remains a concern and most trials evaluate the safety aspect. The pooled risk ratio (RR) of severe adverse events (grade ≥3) was 1.44 (95% CI: 1.17–1.78, *p* = 0.0006, *I*^2^ = 13%) as shown in Figure 8a. The incidence of severe adverse events (AEs) in the oncolytic virus treatment group was higher than the control group (39% vs. 27%), with a pooled risk difference (RD) of severe AEs recorded at 0.12 (95% CI: 0.06–0.18, *p* = 0.0002, *I*^2^ = 37%) (Figure 8b); RD represents the rate of severe AEs attributed to oncolytic virotherapy. Furthermore, we analyzed detailed adverse events that may be associated with oncolytic virus treatment (Table 3). Patients treated with OVs had a higher risk for all-grade AEs such as fever (RR = 3.87, 95% CI: 2.15–6.69, *p* < 0.00001), neutropenia (RR = 1.66, 95% CI:1.21–2.29, *p* = 0.002), diarrhea (RR = 1.56, 95% CI:1.26–1.95, *p* < 0.0001), nausea (RR = 1.49, 95% CI: 1.28–1.74, *p* < 0.00001), vomiting (RR = 1.65, 95% CI: 1.27–2.14, *p* = 0.0002), chills (RR = 7.04, 95% CI: 4.64–10.66, *p* < 0.00001), flu-like symptoms (RR = 4.13, 95% CI:2.15–7.94, *p* < 0.0001), arthralgia (RR = 1.51, 95% CI: 1.09–2.12, *p* = 0.01), myalgia (RR = 1.97, 95% CI: 1.32–2.96, *p* = 0.001), extreme pain (RR = 1.50, 95% CI: 1.06–2.11, *p* = 0.02), headache (RR = 1.90, 95% CI: 1.42–2.53, *p* < 0.0001), and thrombocytopenia (RR = 2.74, 95% CI: 1.65–4.57, *p* = 0.0001). However, only neutropenia treatment yielded statistically significant severe adverse events (RR = 1.36, 95% CI: 1.03–1.80, *p* = 0.03).

#### 3.3.4. Publication Bias and Sensitivity Analysis

**Figure 9:**
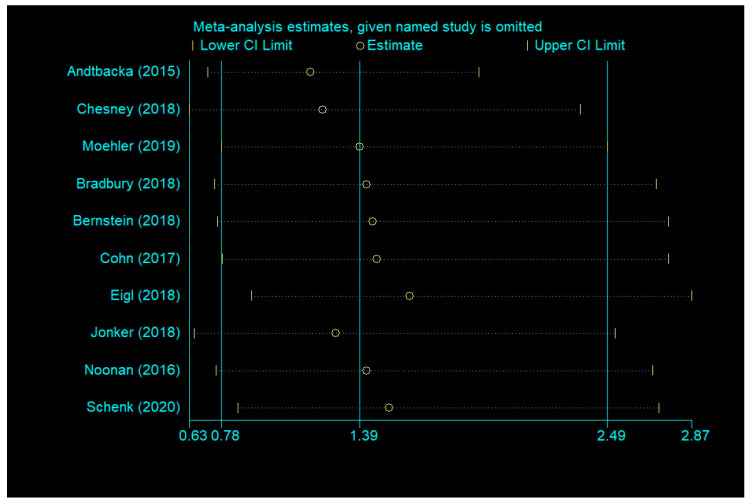
Sensitivity analysis of ORR.

Publication bias was formally assessed using Begg’s test and Egger’s test. OS (Begg’s test, *p* = 0.283; Egger’s test, *p* = 0.126), PFS (Begg’s test, *p* = 0.548; Egger’s test, *p* = 0.307), and severe AEs (Begg’s test, *p* = 0.707; Egger’s test, *p* = 0.966) did not reveal any significant publication bias, but ORR (Begg’s test, *p* = 0.118; Egger’s test, *p* = 0.046 <0.1) had significant differences of publication bias. We made a sensitivity analysis by omitting a study to estimate meta-analysis of ORR. It suggested that omitting any one study had little effect on the overall result (each offset is minimal and between the upper CL limit and lower CL limits) (Figure 9). Therefore, the publication bias of ORR had limited impact on our conclusions.

## 4. Discussion

Oncolytic viruses possess the potential to kill cancerous cells (oncolysis); they also induce antitumor immune response through multiple mechanisms [42,43]. Such characteristics have made oncolytic virotherapy a promising immunotherapeutic approach for cancer patients. However, clinical trials have revealed that the presence of neutralizing antibodies in the blood prevents the oncolytic viruses (except reovirus) from replicating; activation of the immune system leads to rapid elimination of oncolytic viruses, and oncolytic viruses cannot target tumors due to physical parameters [5,44,45]. Furthermore, the best oncolytic virus, route of administration, prognosis of patients, and adverse reactions remain controversial. 

In this study, we extracted data for objective response rate (ORR), overall survival (OS), and progression-free survival (PFS) for in-depth analysis of the effectiveness of oncolytic virotherapy. Generally, T-VEC (OR = 4.05, 95% CI: 1.96–8.33) showed remarkable clinical efficacy of ORR. Interestingly, the objective response rate benefit was observed in patients treated with oncolytic DNA viruses (OR = 4.05, 95% CI: 1.96–8.33) but not in those treated with oncolytic RNA viruses (OR = 1.00, 95% CI: 0.66–1.52). This may be because DNA viruses carry many external genes with important immunomodulatory effects. In addition, DNA viruses express high fidelity DNA polymerases, which maintain the integrity of the viral genome and sufficient amplification [16,43]. Increasing evidence suggests that the antitumor effect of oncolytic viruses is not only dependent on pure oncolysis but also virus-induced antitumor immunity [16,46,47]. The three mechanisms in which oncolytic virus breaks the immune tolerance include: (1) after the virus infects tumor cells, it induces antigen-presenting cells (APCs) to infiltrate the tumor infection site; (2) the tumor antigen released after the virus lyses tumor cells and enhances the antigen presentation ability of APCs, thereby generating a specific immune response against the tumor antigen, forming a long-term antitumor immune response; (3) while OVs replicate in the tumor, they also express immunomodulatory factors, and they jointly participate in further amplification of antitumor immunity [48,49]. Since RNA viruses often replicate quickly and only possess few foreign genes [16,23], their antitumor effect is mainly dependent on oncolysis than immune activation. In respect of injection mode, cancer patients gained a significant objective response rate benefit from intratumoral injection (OR = 4.05, 95% CI: 1.96–8.33). Due to physical parameters and virus dilution, the targeting and effect of intravenous injection were unsatisfactory [5]. Although intratumoral injection can circumvent the above-mentioned problems, it is also limited by tumor type. 

From the survival data, only T-VEC (HR = 0.79, 95% CI: 0.63–0.99, *p* = 0.04) could effectively prolong overall survival (OS) of cancer patients. Pelareorep, Pexa-Vec, and NTX-010 were not statistically significant for OS. Moreover, no oncolytic virus affected progression-free survival (PFS) (HR = 1.00, 95% CI: 0.85–1.19). In patients with metastatic breast cancer, the median survival time of the experimental group (17.4 months) treated with pelareorep was remarkably longer than that of the control group (10.4 months). The HR of overall survival was 0.65 (80% CI: 0.46–0.91, *p* = 0.10). This suggests that pelareorep may be a new promising drug for metastatic breast cancer; more RCTs are, however, needed to validate it. 

Oncolytic viruses are generally considered safe. However, the oncolytic virotherapies were associated with specific risks in this meta-analysis. The pooled risk ratios (RR) and risk difference (RD) of severe adverse events (AEs) were 1.44 (95% CI: 1.17–1.78, *p* = 0.0006) and 0.12 (95% CI: 0.06–0.18, *p* = 0.0002), respectively, indicating such therapies carry risks that should not be ignored. Any-grade AEs with an incidence greater than 10% included fever (48.90%), neutropenia (63.01%), febrile neutropenia (25.18%), leukopenia (71.23%), diarrhea (28.78%), nausea (45.24%), vomiting (27.84%), chills (45.84%), fatigue (55.35%), flu-like symptoms (31.29%), decreased appetite/anorexia (25.91%), arthralgia (19.01%), myalgia (18.42%), extreme pain (20.98%), headache (24.11%), cough (21.66%), and thrombocytopenia (54.79%). Severe AEs with an incidence greater than 5% included neutropenia (40.36%), febrile neutropenia (15.52%) leukopenia (26.61%), fatigue (6.836%), and thrombocytopenia (10.09%). In the one-sided test, statistically significance of high-grade flu-like symptoms (1.23%), cellulitis (5.822%) of any-grade, and decreased appetite/anorexia (25.91%) of any-grade were observed. Detailed severe AEs have not been reported yet, and may be due to the loss of follow up, leading to underestimation.

Our meta-analysis had the following limitations. First, we did not consider tumor types because of the insufficient number of RCTs to analyze same cancer. Secondly, in the subgroup analysis of objective response rate, there were few RCTs about oncolytic DNA viruses and intratumoral injection, and the conclusion needs more research to verify. Besides, the effective oncolytic virus was T-VEC. Therefore, the analysis results of the objective response rate may be affected by it. Finally, the heterogeneity of adverse events was biased upward since a wide range of oncolytic viruses was included. This review may provide new ideas for further research on oncolytic viruses to address the remaining challenges. We believe that oncolytic virotherapy will play an increasingly important role in cancer therapy with the increase of number of studies conducted.

## 5. Conclusions

In conclusion, the results of our meta-analysis showed that the objective response rate benefit was observed in oncolytic DNA viruses and intratumoral injections. Currently, only patients treated with T-VEC can prolong overall survival. Besides, our meta-analysis revealed that occurrence of severe adverse events associated with oncolytic virotherapy cannot be ignored. More qualitative RCTs are needed to test the efficacy and safety of oncolytic viruses.

## Figures and Tables

**Table 1:** Characteristics of the RCTs included in this meta-analysis.

First Author (Year)	Tumor Type	Treatment Arm	Injection Mode	Age (Years)	Male, No. (%)
Andtbacka 2015 [26]	Melanoma	T-VEC vs. GM-CSF	IT	EG: median 63 (22–94) CG: median 64 (26–91)	EG: 173/295 (59%) CG: 77/141 (55%)
Bernstein 2018 [28]	Breast cancer	Pelareorep + paclitaxel vs. paclitaxel	IV	EG: median 61 (44–78) CG: median 57 (36–73)	EG: 0/36 (0%) CG: 0/38 (0%)
Bradbury 2018 [29]	Non-small cell lung cancer	Pelareorep + chemotherapy vs. chemotherapy	IV	EG-1: median 63 (43–78) EG-2: 64 (23–77) CG-1: median 65 (39–80) CG-2: 64 (41–84)	EG: 36/77 (47%) CG: 41/75 (55%)
Chesney 2018 [27]	Melanoma	T-VEC + ipilimumab vs. ipilimumab	IT	EG: median 65 (23–93) CG: median 64 (23–90)	EG: 62/98 (63%) CG: 55/100 (55%)
Cohn 2017 [30]	Ovarian, tubal, or peritoneal cancer	Pelareorep + paclitaxel vs. paclitaxel	IV	NR	EG: 0/54 (0%) CG: 0/54 (0%)
Eigl 2018 [31]	Prostate cancer	Pelareorep + docetaxel vs. docetaxel	IV	EG: median 69.1 (50.3–83.7) CG: median 68.6 (49.7–86.6)	EG: 21/21 (100%) CG: 23/23 (100%)
Freytag 2014 [34]	Prostate cancer	Ad5-yCD/*mut*TK_SR39_*rep*-ADP + IMRT vs. IMRT	IT	EG: mean 68.0 (55–78) CG: mean 65.2 (51–79)	EG: 41/41 (100%) CG: 44/44 (100%)
Jonker 2018 [32]	Colorectal cancer	Pelareorep + FOLFOX6/bevacizumab vs. FOLFOX6/bevacizumab	IV	EG: median 60 (34–79) CG: median 59 (31–78)	EG: 19/51 (37%) CG: 21/52 (40%)
Moehler 2019 [35]	Hepatocellular carcinoma	Pexa-Vec + BSC vs. BSC	IV	EG: mean 60 ± 11 CG: mean 55 ± 12	EG: 72/86 (84%) CG: 33/43 (77%)
Noonan 2016 [33]	Pancreatic adenocarcinoma	Pelareorep + paclitaxel/carboplatin vs. paclitaxel/carboplatin	IV	EG: median 61.5 (39–84) CG: median 66 (45–81)	EG: 22/36 (61.1%) CG: 19/37 (51.4%)
Schenk 2020 [19]	Small cell lung cancer	NTX-010 vs. placebo	IV	EG: median 67 (44–81) CG: median 60 (50–82)	EG: 14/26 (53.9%) CG:10/24 (41.7%)

EG, experimental group; CG, control group; NR, not reported; BSC, best supportive care; IMRT, intensity modulated radiation therapy; IT, intratumoral; IV, intravenous.

**Table 2:** Summary of outcomes in the selected RCTs.

First Author (Year)	Median OS (Months)	HR (95% CI) for OS	Median PFS (Months)	HR (95% CI) for PFS	ORR	Severe Adverse Event
Andtbacka 2015 [26]	EG: 23.3 CG: 18.9	0.79 (0.62, 1.00)	NR	NR	EG: 78 CG: 8	EG:105 CG: 27
Bernstein 2018 [28]	EG: 17.4 CG: 10.4	0.61 (0.33, 1.12)	EG: 3.78 CG: 3.38	1.11 (0.64, 1.92)	EG: 9 CG: 9	EG: 18 CG: 18
Bradbury 2018 [29]	EG: 7.8 CG: 7.4	0.98 (0.72, 1.34)	EG: 3.0 CG: 2.8	0.90 (0.65, 1.25)	EG: 11 CG: 11	NR
Chesney 2018 [27]	NR	0.80 (0.44, 1.46)	EG: 8.2 CG: 6.4	0.83 (0.56, 1.23)	EG: 38 CG: 18	EG: 43 CG: 33
Cohn 2017 [30]	EG: 12.6 CG: 13.1	1.01 (0.64, 1.58)	EG: 4.4 CG: 4.3	1.11 (0.64, 1.91)	EG: 8 CG: 9	NR
Eigl 2018 [31]	NR	1.86 (0.97, 3.57)	NR	NR	EG: 11 CG: 18	NR
Freytag 2014 [34]	No death	NR	No death	NR	NR	EG: 1 CG: 1
Jonker 2018 [32]	EG: 19.2 CG: 20.1	1.18 (0.75, 1.87)	EG: 7.33 CG: 9.13	1.65 (1.02, 2.67)	EG: 27 CG: 18	NR
Moehler 2019 [35]	EG: 4.2 CG: 4.4	1.19 (0.77, 1.83)	EG: 4.94 CG: 5.2	NR	EG: 0 CG: 0	EG: 45 CG: 7
Noonan 2016 [33]	EG: 7.31 CG: 8.77	1.12 (0.66, 1.91)	EG: 1.7 CG: 1.7	0.86 (0.52, 1.43)	EG: 7 CG: 7	NR
Schenk 2020 [19]	EG: 6.6 CG: 13.2	1.49 (0.77, 2.87)	NR	1.03 (0.58, 1.83)	EG: 1 CG: 4	EG: 9 CG: 5

EG, experimental group; CG, control group; HR, hazard ratio; OS, overall survival; PFS, progression-free survival; NR, not reported; CI, confidence interval.

**Table 3:** Adverse events of interest.

Adverse Event	All Grades				Grade ≥3			
	*I* ^2^	RR (95% CI)	*p*	Incidence of EG	*I* ^2^	RR (95% CI)	*p*	Incidence of EG
Fever	73%	3.87 (2.15, 6.69)	<0.00001 *	48.90%	0%	3.07 (0.62, 15.10)	0.17	1.825%
Neutropenia	67%	1.66 (1.21, 2.29)	0.002 *	63.01%	50%	1.36 (1.03, 1.80)	0.03 *	40.36%
Febrile neutropenia	66%	1.76 (0.66,4.69)	0.25	25.18%	3%	1.19 (0.77, 1.84)	0.44	15.52%
Leukopenia	36%	1.21 (0.96, 1.51)	0.11	71.23%	90%	1.84 (0.23, 14.36)	0.56	26.61%
Diarrhea	17%	1.56 (1.26, 1.95)	<0.0001 *	28.78%	13%	1.12 (0.56, 2.22)	0.75	2.178%
Nausea	35%	1.49 (1.28, 1.74)	<0.00001 *	45.24%	0%	1.05 (0.48, 2.29)	0.89	1.754%
Vomiting	36%	1.65 (1.27, 2.14)	0.0002 *	27.84%	5%	0.68 (0.30, 1.52)	0.35	1.983%
Chills	32%	7.04 (4.64, 10.66)	<0.00001 *	45.84%	NA	0.92 (0.04, 21.85)	0.96	0.1825%
Fatigue	85%	1.22 (0.95, 1.57)	0.12	55.35%	0%	1.24 (0.83, 1.85)	0.29	6.836%
Flu-like symptoms	60%	4.13 (2.15, 7.94)	<0.0001 *	31.29%	0%	4.41 (0.82, 23.81)	0.08	1.23%
Decreased appetite/anorexi-a	25%	1.23 (0.98, 1.56)	0.08	25.91%	51%	0.55 (0.17, 1.76)	0.32	0.6048%
Arthralgia	13%	1.51 (1.09, 2.12)	0.01 *	19.01%	0%	0.94 (0.19, 4.67)	0.94	0.6073%
Myalgia	47%	1.97 (1.32, 2.96)	0.001 *	18.42%	NA	1.31 (0.05, 31.96)	0.87	0.2208%
Pain in extremity	0%	1.50 (1.06, 2.11)	0.02 *	20.98%	0%	1.57 (0.40, 6.21)	0.52	1.897%
Headache	0%	1.90 (1.42, 2.53)	<0.0001 *	24.11%	0%	1.86 (0.47, 7.34)	0.38	1.095%
Cough	17%	0.85 (0.67, 1.07)	0.17	21.66%	NA	0.32 (0.01, 7.85)	0.49	0
Cellulitis	NA	3.70 (0.87, 15.76)	0.08	5.822%	NA	2.64 (0.31, 22.18)	0.37	2.055%
Thrombocytope-nia	0%	2.74 (1.65, 4.57)	0.0001 *	54.79%	0%	1.23 (0.58, 2.61)	0.59	10.09%

*, statistically significant value; 95% CI, 95% confidence interval; RR, risk ratio; NA, not available; *I*^2^, index of heterogeneity; EG, experimental group.
